# Provisional stenting with side branch rescue stenting is associated with increased 3-year target lesion failure in patients with acute coronary syndrome and coronary bifurcation lesions

**DOI:** 10.3389/fcvm.2022.910313

**Published:** 2022-10-11

**Authors:** Imad Sheiban, Zhen Ge, Jing Kan, Jun-Jie Zhang, Teguh Santoso, Muhammad Munawar, Fei Ye, Nailiang Tian, Shao-Liang Chen

**Affiliations:** ^1^Division of Cardiology, Pederzoli Hospital-Peschiera del Garda, Verona, Italy; ^2^Division of Cardiology, Nanjing First Hospital, Nanjing Medical University, Nanjing, China; ^3^Division of Cardiology, Medistra Hospital, University of Indonesia Medical School, Jakarta, Indonesia; ^4^Division of Cardiology, Binawaluya Cardiac Center, Jakarta, Indonesia

**Keywords:** acute coronary syndrome (ACS), coronary artery bifurcation lesions, provisional stenting, drug-eluting stent, target lesion failure

## Abstract

**Background:**

Provisional stenting (PS) is the main treatment for a majority of coronary bifurcation lesion and includes PS with 1-stent and PS with 2-stent. However, the treatment difference between PS with 1-stent and with 2-stent remains unclear in patients with the acute coronary syndrome (ACS) and coronary bifurcation lesions.

**Materials and methods:**

Overall, 820 ACS patients with Medina 1,1,1 or 0,1,1 coronary bifurcation lesion who had completed 3-year follow-up were included and assigned to the PS with 1-stent (n = 519) or the PS with 2-stent (n = 301) according to the use of final stenting technique. The primary endpoint was the target lesion failure (TLF) at 3 years since stenting procedures.

**Results:**

At 3-year follow-up, TLF occurred in 85 (16.4%) patients in the PS with 1-stent group and 69 (22.9%) in the PS with 2-stent group (hazard ratio [HR] 1.52, 95% confidence interval [CI] 1.06–2.17, p = 0.021), mainly driven by a higher rate of target lesion revascularization (TLR) in the PS with 2-stent group (13.0% vs. 8.3%, HR 1.65, 95% CI 1.04–2.61, p = 0.033). Complex bifurcations, side branch (SB) pretreatment, intravascular imaging guidance, and hyperlipidemia were the four predictors for 3-year TLF. SB pretreatment was associated with increased 3-year TLR, leading to an extremely higher 3-year TLF.

**Conclusion:**

Provisional with 2-stent for patients with ACS is associated with a higher rate of 3-year TLF, mainly due to increased requirement of revascularization. SB pretreatment should be avoided for simple bifurcation lesion.

## Graphical Abstract

Landmark analysis of target lesion failure and target lesion revascularization. 
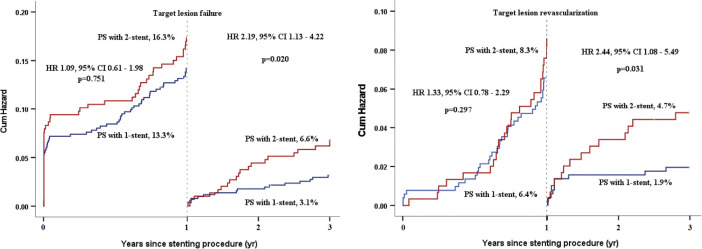

## Introduction

Coronary artery bifurcation lesions involve three vessel segments (proximal main vessel [MV], distal MV, and side branch [SB]), leading to technical challenging of bifurcation stenting and suboptimal clinical outcomes (1, 2). While upfront two-stent approach (like DK crush stenting) has been demonstrated to be associated with less rate of target lesion failure (TLF) for the treatment of patients with bifurcation lesions localizing at distal left main (LM) or having higher complexity (3, 4), provisional stenting (PS) is still accepted to be a major technique for simple bifurcation lesions (2, 5–7). PS requires a jailed wire or balloon in the SB, which could rescue an SB at risk of occlusion after stenting MV. Thus, PS could be shifted to PS with 1-stent or PS with 2-stent, with a rate of crossover to 2-stent varying from 2 to 40% (2, 4–9), depending on the performance of SB pretreatment and final kissing balloon inflation (KBI), lesions’ complexity, flow-limiting dissection, severely compromised ostial SB induced by plaque or carina shifting, and criteria for treating SB in clinical trials. As a result, PS with SB rescue stenting is unavoidable for complex bifurcations (4, 9). However, there is a paucity of data showing the difference in clinical outcomes between PS with 1-stent and PS with 2-stent among patients with acute coronary syndrome (ACS) and bifurcation lesions. Accordingly, this study includes all ACS patients with bifurcations who underwent the PS approach and had completed 3-year clinical follow-up from previous four trials (4, 9–11) with a view to identify the rate of crossover to 2-stent, the difference in 3-year TLF between PS with 1-stent and with 2-stent, and the independent factors of 3-year TLF.

## Materials and methods

### Study design

**FIGURE 1:**
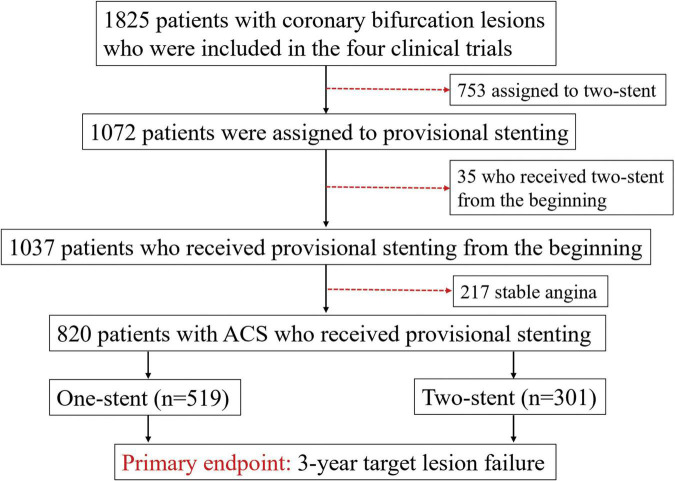
Study flowchart. Description: 820 patients with acute coronary syndrome (ACS) were assigned to the provisional stenting (PS) with 1-stent and the PS with 2-stent groups.

We included data of the following clinical trials with only Medina 1,1,1 and 0,1,1 bifurcation lesions in patients with ACS: DKCRUSH II (10), DKCRUSH V (9), DKCRUSH VI (11), and DEFINITION II (4; Figure 1). All patients were prospectively followed up till January 1, 2022. The study protocol was approved by the ethics committee at each participating center and complied with the Declaration of Helsinki. All patients provided written informed consent for participation in the respective trials. All authors had free access to the database.

### Patient population

Overall, 1,825 patients with coronary bifurcation lesions from the four clinical trials were screened. We excluded 1,005 patients because of upfront 2-stent (*n* = 753) at randomization, SB pretreatment leading to the urgent requirement of stenting SB before stenting the MV (*n* = 35), and stable angina patients (*n* = 217). Finally, 820 patients with ACS who underwent PS were included (Figure 1), with 519 patients assigned to the PS with 1-stent group and 301 patients assigned to the PS with 2-stent group.

### Provisional stenting procedures

The PS approach has been previously described (4, 9–11). In brief, the MV and SB are wired. Predilation was left to the operator’s discretion, although predilating the SB is discouraged. A new-generation drug-eluting stent was used in the bifurcation lesions. A stent with a stent/artery ratio of 1.1:1 was implanted in the MV, and then the proximal optimization technique (POT) using non-compliant balloons (balloon/stent ratio of 1:1, > 18 atm) was performed. Ballooning or stenting the SB after MV stenting is performed only if the SB ostium is severely compromised or has a Type B/C dissection or thrombolysis in myocardial infarction (TIMI) flow < 3. If SB dilatation or stenting is required, the SB is rewired through a distal cell of the MV stent, followed by re-POT, KBI, and final POT using non-compliant balloons with a suggested inflation pressure of >18 atm.

### Medications and follow-up

All patients were treated with aspirin preprocedure and 300 mg loading dose of clopidogrel or 180 mg ticagrelor if they were not under chronic dual antiplatelet therapy (DAPT). After the intervention, they received 100 mg/day aspirin indefinitely and 75 mg/day clopidogrel or 180 mg (90 mg, bid) ticagrelor for at least 12 months. A clinical follow-up was performed at 1 and 12 months and annually subsequently through 3 years.

Follow-up coronary angiography was scheduled at 13 months (after ascertainment of the primary clinical endpoint) unless performed earlier for clinical indications. Quantitative coronary analysis (QCA) was analyzed at a central core laboratory using the Cardiovascular Angiographic Analysis System (CAAS) II software (Pie Medical Imaging, The Netherlands), as previously described (4). Restenosis was defined as a QCA DS > 50% at follow-up.

### Endpoints and definitions

The primary endpoint was TLF at 3 years, defined as the composite of cardiac death, target vessel MI (TVMI), or clinically driven TLR. Death from cardiac causes was defined as any death without a clear non-cardiac cause. Protocol-defined periprocedural MI (within 48 h) was defined as a creatine kinase-MB (CK-MB) > 10 × upper reference limit (URL) of the assay or > 5 × URL plus either (1) new pathological Q waves in ≥ 2 contiguous leads or new left bundle branch block (LBBB); (2) angiographically documented graft or coronary artery occlusion or new severe stenosis with thrombosis; (3) imaging evidence of new loss of viable myocardium; or (4) new regional wall motion abnormality. Spontaneous MI (after 48 h) was defined as a clinical syndrome consistent with MI with a CK-MB or troponin > 1 × URL and new ST-segment elevation or depression or other findings as above. All MIs were considered TVMI unless there was clear evidence that they were attributable to a non-target vessel (4, 12). Clinically driven TLR was defined as angina or ischemia referable to the target lesion requiring repeat PCI or coronary artery bypass graft. Secondary endpoints included cardiac death, TVMI, clinically driven TLR, and stent thrombus (ST). Definite or probable ST according to the Academic Research Consortium (13) was the major safety endpoint. All events were adjudicated by a central committee using original source documents blinded to the treatment.

### Statistical analysis

Baseline characteristics are reported as counts and percentages or mean ± standard deviation (SD). The chi-squared or Fisher’s exact test was used to compare categorical variables. Student’s *t*-test or Wilcoxon rank-sum scores for non-normally distributed data were used to compare continuous variables. Time-to-first event curves were generated using Kaplan–Meier analysis and compared using the log-rank test. Cox regression was also used to compare the differences in both primary and secondary endpoints, with outputs of hazard ratio (HR), 95% confidence interval (CI), and *p*-value. Multivariate analysis was performed to identify the independent factors of 3-year TLF. All statistical tests were two-sided, and a *p*-value of <0.05 was considered statistically significant. All analyses were performed using SPSS version 26.0 (SPSS Institute Inc., Chicago, Illinois, USA).

## Results

### Baseline clinical characteristics

Baseline clinical characteristics were well comparable between the groups (Table 1), except for unstable angina (85.0% in the 2-stent group vs. 75.3% in the 1-stent group, *p* = 0.001) and ST-segment elevation MI (4.7% in the 2-stent group vs. 12.1% in the 1-stent group, *p* < 0.001). Diabetes was present in 27.6% of patients.

**TABLE 1:** Baseline characteristics.

	PS with 1-stent (*n* = 519)	PS with 2-stent (*n* = 301)	*P*-value
Age, year	64.6 ± 9.8	64.4 ± 10.1	0.721
Male, n (%)	399 (76.9)	228 (75.7)	0.733
Hypertension, n (%)	343 (66.1)	203 (67.4)	0.702
Systolic blood pressure, mmHg	133 ± 16	134 ± 18	0.384
Diastolic blood pressure, mmHg	79 ± 10	79 ± 10	1.000
Heart rate, beats per minute	73 ± 12	73 ± 10	0.706
Hyperlipidemia, n (%)	214 (41.2)	111 (36.9)	0.236
Diabetes, n (%)	147 (28.3)	81 (26.9)	0.687
Current smoker, n (%)	101 (19.6)	63 (21.1)	0.601
Renal dysfunction, n (%)	15 (2.9)	10 (3.3)	0.834
Previous PCI, n (%)	92 (17.7)	55 (18.3)	0.851
Previous CABG, n (%)	3 (0.6)	1 (0.3)	1.000
Previous MI, n (%)	69 (13.3)	50 (16.6)	0.217
Stroke, n (%)	45 (8.7)	22 (7.3)	0.597
Peripheral arterial disease, n (%)	21 (4.0)	16 (5.3)	0.390
Heart failure, n (%)	58 (11.2)	37 (12.3)	0.651
Atrial fibrillation, n (%)	10 (1.9)	8 (2.7)	0.623
eGFR < 60 ml/min/1.73m^2^	77 (14.8)	37 (12.3)	0.310
Presentation, n (%)			
Unstable angina	388 (75.3)	256 (85.0)	0.001
STEMI > 24 h	63 (12.1)	14 (4.7)	<0.001
NSTEMI > 24 h	68 (13.1)	31 (10.3)	0.235

PS, provisional stenting; PCI, percutaneous coronary intervention; CABG, coronary artery bypass graft; MI, myocardial infarction; eGFR, estimated glomerular filtration rate; STEMI, ST-segment elevation myocardial infarction; NSTEMI, ST-segment elevation myocardial infarction.

### Lesion characteristics and procedures

Multivessel disease was present in 52.2% of patients, and the mean SYNTAX score was 26 (Table 2). Notably, 37.4% of the lesions were localized in the distal LM. Complex bifurcation lesions were seen in 59.1% of patients in the 2-stent group, compared to 43.0% in the 1-stent group (*p* < 0.001), with an extremely higher rate of SB lesion length ≥ 10 mm in the 2-stent group (42.9% vs. 32.2%, *p* = 0.002), as confirmed by the QCA analysis (Table 3).

**TABLE 2:** Lesions and procedural characteristics.

	PS with 1-stent (*n* = 519)	PS with 2-stent (*n* = 301)	*P*-value
Multiple vessel disease, n (%)	282 (54.3)	151 (50.2)	0.276
SYNTAX Score, scores	25.68 ± 10.9	26.27 ± 11.2	0.458
≤22 scores, n (%)	213 (41.0)	117 (38.9)	0.541
23∼32 scores, n (%)	167 (32.3)	95 (31.6)	0.855
≥32 scores, n (%)	139 (26.8)	89 (29.6)	0.391
Lesion location, n (%)			0.429
LAD-LCX	178 (34.3)	122 (40.5)	
LAD-D	265 (51.1)	140 (46.5)	
LCX-OM	54 (10.4)	28 (9.3)	
Distal RCA	22 (4.2)	11 (3.7)	
True bifurcation lesions, n (%)	464 (89.6)	280 (93.0)	0.104
Complex bifurcation lesion, n (%)	223 (43.0)	178 (59.1)	<0.001
No. lesion, n	2.20 ± 0.91	2.24 ± 0.95	0.642
No. treated lesion, n	1.96 ± 0.81	2.02 ± 0.86	0.481
SB lesion length ≥ 10 mm, n (%)	167 (32.2)	129 (42.9)	0.002
≥Moderate calcification, n (%)	156 (30.1)	85 (28.2)	0.633
Chronic total occlusion, n (%)	37 (7.1)	32 (10.6)	0.119
Thrombus-containing lesion, n (%)	22 (4.2)	6 (2.0)	0.110
TIMI flow < 3 prior-to PCI, n (%)			
Main vessel	99 (9.1)	62 (20.6)	0.172
Side branch	40 (7.7)	27 (9.0)	0.859
Trans-radial approach, n (%)	368 (83.1)	216 (82.8)	0.427
MV pretreatment, n (%)	216 (41.6)	59 (19.6)	<0.001
SB pretreatment, n (%)	167 (32.2)	171 (56.8)	<0.001
IVUS guidance, n (%)	123 (23.7)	66 (21.9)	0.606
MV stent			
No. stent, n	1.65 ± 0.68	1.55 ± 0.67	0.054
Average diameter, mm	3.05 ± 0.61	3.09 ± 0.39	0.327
Average length, mm	43.66 ± 20.09	41.91 ± 21.37	0.241
Proximal optimization technique, n (%)	492 (94.8)	269 (89.4)	0.005
Balloon diameter, mm	3.79 ± 0.61	3.86 ± 0.43	0.689
Inflation pressure, atm	17.97 ± 3.21	17.73 ± 3.99	0.812
Final kissing inflation, n (%)	215 (41.4)	287 (95.3)	<0.001
Complete revascularization, n (%)	322 (62.0)	206 (68.4)	<0.001
Contrast volume, ml	158 ± 79	183 ± 84	<0.001
Procedural time, min	55.8 ± 37.2	65.3 ± 36.0	<0.001

PS, provisional stenting; LAD, left anterior descending artery; LCX, left circumflex; D, diagonal; OM, obtuse marginal; RCA, right coronary artery; SB, side branch; PCI, percutaneous coronary intervention; MV, main vessel; IVUS, intravascular ultrasound.

**TABLE 3:** Quantitative angiographic analysis.

	PS with 1-stent (*n* = 286)	PS with 2-stent (*n* = 141)	*P*-value
MV lesion length, mm	32.34 ± 16.79	27.52 ± 16.58	0.003
Proximal MV	12.66 ± 9.56	10.89 ± 10.32	0.016
Distal MV	19.43 ± 12.92	17.86 ± 14.21	0.118
SB lesion length, mm	12.04 ± 7.75	15.11 ± 7.18	<0.001
Distal bifurcation angle, 0°			
Prior-to	72.2 ± 39.5	82.8 ± 41.8	0.001
Post-stenting	69.5 ± 38.2	77.1 ± 42.3	0.020
Follow-up	73.9 ± 40.3	70.9 ± 40.7	0.462
Proximal MV reference diameter, mm			
Prior-to	3.19 ± 0.49	3.16 ± 0.51	0.385
Post-stenting	3.28 ± 0.49	3.39 ± 0.47	0.002
Follow-up	3.26 ± 0.49	3.29 ± 0.46	0.491
Proximal MV MLD, mm			
Prior-to	1.78 ± 0.82	1.77 ± 0.79	0.888
Post-stenting	2.89 ± 0.55	3.08 ± 0.49	<0.001
Acute gain	1.11 ± 0.79	1.32 ± 0.74	<0.001
Follow-up	2.81 ± 0.58	2.83 ± 0.58	0.658
Late loss	0.11 ± 0.38	0.19 ± 0.39	0.019
Proximal MV diameter stenosis, %			
Prior-to	45.0 ± 22.9	43.6 ± 23.1	0.421
Post-stenting	12.1 ± 9.9	9.3 ± 6.8	<0.001
Follow-up	11.8 ± 9.8	11.4 ± 9.3	0.588
Restenosis, n (%)	3 (1.0)	2 (1.3)	0.667
Distal MV reference diameter, mm			
Prior-to	2.65 ± 0.46	2.69 ± 0.51	0.231
Post-stenting	2.74 ± 0.43	2.82 ± 0.42	0.023
Follow-up	2.77 ± 0.44	2.77 ± 0.40	0.943
Distal MV MLD, mm			
Prior-to	1.21 ± 0.59	1.15 ± 0.63	0.214
Post-stenting	2.37 ± 0.46	2.46 ± 0.43	0.003
Acute gain	1.15 ± 0.61	1.31 ± 0.63	0.001
Follow-up	2.26 ± 0.54	2.23 ± 0.54	0.570
Late loss	0.13 ± 0.38	0.22 ± 0.44	0.013
Distal MV diameter stenosis, %			
Prior-to	53.8 ± 21.4	56.9 ± 22.9	0.050
Post-stenting	14.5 ± 10.8	12.9 ± 8.8	0.033
Follow-up	17.5 ± 14.4	18.6 ± 15.3	0.383
Restenosis, n (%)	12 (4.2)	8 (5.7)	0.244
SB reference diameter, mm			
Prior-to	2.37 ± 0.44	2.47 ± 0.43	0.002
Post-stenting	2.27 ± 0.46	2.58 ± 0.37	<0.001
Follow-up	2.28 ± 0.47	2.50 ± 0.39	<0.001
SB MLD, mm			
Prior-to	1.29 ± 0.39	1.29 ± 0.57	0.997
Post-stenting	1.42 ± 0.59	2.16 ± 0.40	<0.001
Acute gain	0.12 ± 0.54	0.99 ± 0.54	<0.001
Follow-up	1.43 ± 0.59	1.83 ± 0.61	<0.001
Late loss	0.04 ± 0.43	0.29 ± 0.52	<0.001
SB diameter stenosis, %			
Prior-to	44.7 ± 20.6	54.1 ± 18.9	<0.001
Post-stenting	37.2 ± 20.6	13.8 ± 10.1	<0.001
Follow-up	33.8 ± 23.1	24.8 ± 20.9	<0.001
Restenosis, n (%)	90 (31.5)	22 (15.6)	<0.001
Ostial SB	84 (29.4)	19 (13.5)	<0.001

PS, provisional stenting; MV, main vessel; MLD, minimal lumen diameter; SB, side branch.

The trans-radial approach was predominantly used (82%, Table 2). In the 2-stent group, SB pretreatment was used in 56.8%, significantly different from 32.2% in the 1-stent group (*p* < 0.001), resulting in more frequent use of KBI in the 2-stent group (95.3 vs. 41.4%, *p* < 0.001). Final POT was only used in 89.4% of patients in the 2-stent group, lower than 94.8% in the 1-stent group (*p* < 0.001). Complete revascularization was achieved in 68.4% of patients in the 2-stent group, compared to 62.0% in the 1-stent group (*p* < 0.001). IVUS guidance was only used in <30.0% of patients, without a significant difference between the groups. The two-stent strategy was associated with longer procedural time and more contrast volume compared with the PS with 1-stent approach.

### Quantitative coronary analysis

Except for longer SB lesion length, the 2-stent group also had a more severe SB diameter stenosis (54.1 vs. 44.7%, *p* < 0.001) at baseline. At 3 years since procedures, a total of 427 (52.1%) patients underwent repeat angiography, with 246 (30.0%) at 13 months and 181 (22.1%) after 13 months. The in-stent restenosis (ISR) rate in the MV was non-significantly different between the 2 groups (Table 3). In the 2-stent group, the rate of ISR at the ostial SB was 13.3%, compared to 29.4% in the 1-stent group (*p* < 0.001).

### Clinical outcomes

At 3 years, DAPT was prescribed to 203 (39.1%) patients in the PS with 1-stent and 198 (65.8%) in the PS with 2-stent (*p* < 0.001). Ticagrelor (90 mg; twice a day) was administered in 58.6% of patients in the PS with 2-stent group and 38.2% in the PS with 1-stent group (*p* < 0.001).

A 1-year clinical follow-up was completed in all patients. The primary endpoint of TLF at 1 year occurred in 69 (13.3%) patients in the PS with 1-stent group and 49 (16.3%) patients in the PS with 2-stent group (HR 1.09, 95% CI 0.61–1.98, *p* = 0.751) (Table 4). The rates of TVMI, cardiac death, TLR, and ST at 1 year were also non-significantly different between the two groups.

**TABLE 4:** Clinical results.

	PS with 1-stent (*n* = 519)	PS with 2-stent (*n* = 301)	Adjusted		
			
			HR	95% CI	*p*
30 days, n (%)					
TLF	34 (6.6)	24 (8.0)	1.24	0.83–1.85	0.296
Cardiac death	5 (1.0)	1 (0.3)	0.34	0.04–2.95	0.329
TVMI	30 (5.8)	22 (7.3)	1.29	0.73–2.27	0.388
PMI	16 (3.1)	8 (2.7)			
TLR	4 (0.8)	1 (0.3)	0.43	0.05–3.86	0.450
Stent thrombosis	6 (1.2)	5 (1.7)	1.44	0.44–4.77	0.547
1-year, n (%)					
TLF	69 (13.3)	49 (16.3)	1.09	0.61–1.98	0.751
Cardiac death	11 (2.1)	5 (1.7)	0.78	0.27–2.27	0.648
TVMI	33 (6.4)	29 (9.6)	1.57	0.93–2.64	0.089
STEMI	2 (0.4)	4 (1.3)			
TLR	33 (6.4)	25 (8.3)	1.33	0.78–2.29	0.296
Stent thrombosis	11 (2.1)	10 (3.3)	1.59	0.66–3.78	0.297
Definite	3 (0.6)	4 (1.3)			
Probable	8 (1.6)	6 (2.0)			
3-year, n (%)					
TLF	85 (16.4)	69 (22.9)	1.52	1.06–2.17	0.021
Cardiac death	19 (3.7)	12 (4.0)	1.09	0.52–2.28	0.814
TVMI	41 (7.9)	36 (12.0)	1.58	0.99–2.54	0.056
TLR	43 (8.3)	39 (13.0)	1.65	1.04–2.61	0.033
Stent thrombosis	16 (3.1)	16 (5.3)	1.77	0.87–3.58	0.116
Definite	5 (1.0)	6 (1.8)			
Probable	11 (2.1)	9 (2.7)			

PS, provisional stenting; HR, hazard ratio; CI, confidence interval; TLF, target lesion failure; TVMI, target vessel myocardial infarction; PMI, periprocedural myocardial infarction; TLR, target lesion revascularization.

**FIGURE 2:**
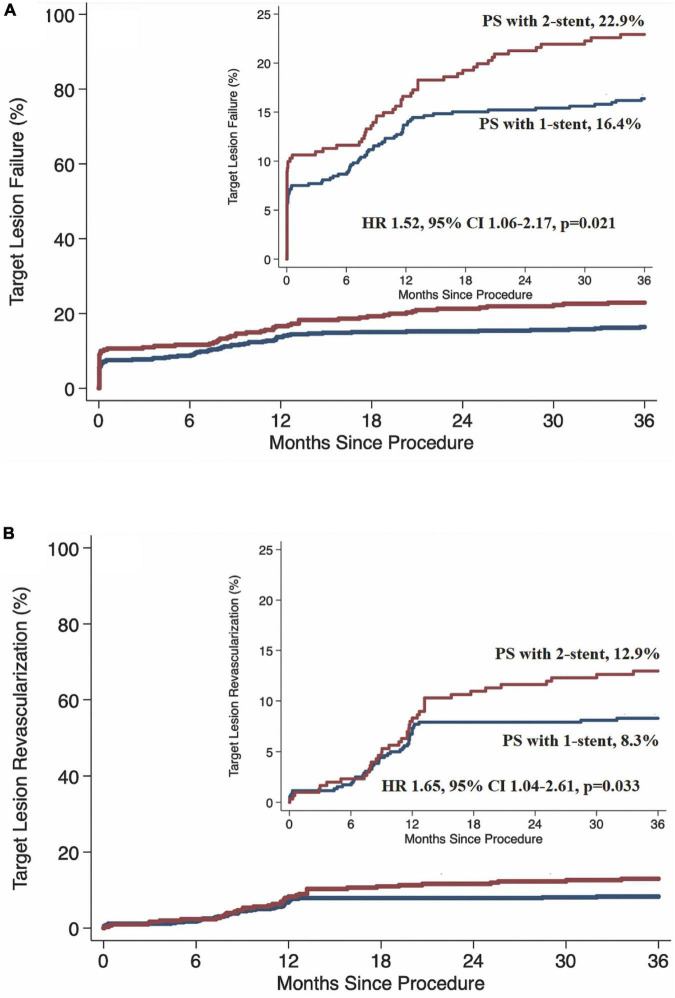
Kaplan–Meier survival rate. PS, provisional stenting; HR, hazard ratio; CI, confidence interval. **(A)** Target lesion failure. **(B)** Target lesion revascularization.

At 3 years, 12 (1.6%) patients were lost to the follow-up, with 7 (2.3%) in the PS with 2-stent group and 5 (0.9%) in the PS with 1-stent group. TLF at 3 years occurred in 85 (16.4%) patients in the PS with 1-stent group and 69 (22.9%) in the PS with 2-stent group (HR 1.52, 95% CI 1.06–2.17, *p* = 0.021, Table 4 and Figure 2A), mainly driven by increased TLR (8.3% vs. 13.0%, HR 1.65, 95% CI 1.04–2.61, *p* = 0.033, Table 4 and Figure 2B), without statistical differences in cardiac death, TVMI, and ST. By landmark analysis (Graphic Abstract), the rates of TLF and TLR at 1 year were comparable between PS with 1-stent and PS with 2-stent; however, the increased rate of TLR from year 1 to year 3 was 3.5% in the PS with 2-stent group (4.7 vs. 1.9%, HR 2.44, 95% CI 1.08–5.49, *p* = 0.031, Graphic Abstract), resulted in a significant difference in TLF after 1 year between the PS with 2-stent (6.6%) and the PS with 1-stent (3.1%, HR 2.19, 95% CI 1.13–4.22, *p* = 0.020).

By multivariate analysis, SB pretreatment (HR 1.66, 95% CI 1.19–2.31, *p* = 0.003), complex bifurcation lesions (HR 2.76, 95% CI 1.42–5.38, *p* = 0.003), without intravascular imaging (HR 1.51, 95% CI 1.07–2.14, *p* = 0.020), and hyperlipidemia (HR 1.15, 95% CI 1.04–1.88, *p* = 0.006) were the four independent factors of 3-year TLF. SB pretreatment was performed because operators were worried about the abrupt closure of an SB after ballooning or stenting MV. Supplementary Table 1 shows that the SB pretreatment subgroup had a longer lesion length (mean length of 14.47 vs. 12.25 mm, *p* = 0.001) and more severe disease (mean diameter stenosis of 51.99 vs. 42.96%) in the SB compared to those in the non-pretreatment subgroup. Subsequent clinical follow-up demonstrated a higher rate of 30-day PMI (8.6%) in the SB pretreatment subgroup, compared to 4.8% (*p* = 0.036) in the non-pretreatment subgroup. At 1- and 3-year follow-up, the increased rates of TVMI and TLR in the PS with 2-stent group led to significantly different TLF rates in the pretreatment subgroup (18.9 and 24.6%) when compared with 11.2% (*p* = 0.002) and 14.7% (*p* = 0.001) in the non-pretreatment subgroup, respectively (Supplementary Figure 1).

## Discussion

This analysis for the first time compared the difference in clinical outcome between the PS with 1-stent and the PS with 2-stent for patients with ACS. The major findings are (1) PS with 2-stent is associated with a higher rate of TLF at 3-year follow-up, largely driven by an increased 3-year rate of TLR, compared to those in the PS with 1-stent group; (2) by multivariate analysis, SB pretreatment, complex bifurcation lesions, without intravascular imaging, and hyperlipidemia were the four independent factors of 3-year TLF; and (3) the rate of TLF at 3-year follow-up in the SB pretreatment subgroup is significantly higher than that in the no pretreatment subgroup, mainly induced by the extreme increments in TVMI and TLR.

The PS with 2-stent is a rescue strategy for SB and reduces the incidence of SB occlusion and PMI and is required in 2–40% of bifurcation lesion treated by provisional approach (2, 4–9). This wide discrepancy in the rate of cross-over to two stents is due to the different criteria for treating SB from previous trials (4–9). For example, SB TIMI flow < 3 was the only criterion for stenting SB in the BBC ONE trial (6), and composite criteria of TIMI flow < 3, Type B or C dissection, and severely compromised in the SB after stenting MV were more commonly used in others (2–5, 7, 9–11). While a few risk stratifications have been proposed to predict the occurrence of clinical events after the PS or upfront two-stent techniques (3, 14–16), the difference in treatment effect between the PS with 1-stent and the PS with 2-stent remained understudied. To echo this issue, Song et al. (17, 18) for the first time randomized 258 patients with a coronary bifurcation lesion to the conservative and aggressive groups according to the criteria for SB intervention after MV stenting (for non-LM bifurcations, the criterion for SB treatment was TIMI < 3 [conservative] or diameter stenosis > 75% [aggressive]; for LM bifurcation lesions, the criterion for SB treatment was diameter stenosis > 75% [conservative] or > 50% [aggressive]). The study reported that at a 3-year follow-up, the primary endpoint (target vessel failure [TVF]) occurred in 11.7% of the conservative group vs. 20.8% of the aggressive group (*p* = 0.049). Although no significant differences were observed in the incidence of TVF between groups at 1 year, landmark analysis between 1 and 3 years showed significantly less TVF in patients assigned to the conservative strategy (2.6 vs. 12.7%; *p* = 0.004). Conservative treatment for SB obviously had a less requirement of additional SB stent. However, the real difference in TVF between the PS with 1-stent and 2-stent could not be directly derived from that study with a small sample size. In this study, we found that a higher rate of 3-year TLF in the PS with 2-stent group was predominantly induced by the increased requirement of TLR after 1-year follow-up, which indicated the reduction in the durability of the second stent in the SB. While the crossover to the 2-stent technique was an independent predictor of TVF (4, 17, 18), we further found that complex bifurcation lesions, SB pretreatment, IVUS use, and hyperlipidemia were the four predictors of 3-year TLF. In this study, IVUS was only used in less than 30% of procedures; although it was equally used in the two groups, the results still recommended the importance of routine use of IVUS in improving the clinical outcome after the PS approach (19–21). In contrast, complex bifurcation defined by DEFINITION criteria (3, 4, 8) was associated with a higher rate of crossover to two stents and a subsequent increased rate of MI and TLR. As a result, an upfront two-stent approach may be an appropriate option for real complex bifurcation lesions.

Side branch pretreatment mirrors the complexity of a given bifurcation lesion, particularly in the SBs having a higher grade of diameter stenosis, as shown by our data. To answer the correlation of the SB pretreatment with increased TVF, Song et al. (22) reported that an additional SB stent was frequently required in the SB pretreatment group and that SB pretreatment increased the rate of TLR and TVF at 24-month follow-up, similar to our results. Recently, a meta-analysis (23) including the four studies demonstrated that bifurcation lesions stented without SB predilation demonstrated lower OR of requiring further SB intervention compared with lesions receiving upfront SB predilation. In fact, our result showed a prediction of SB pretreatment for PMI. More recently, Sheiban et al. (12) reported that PMI was positively correlated with TLF at 1-year follow-up after stenting bifurcation lesions. Conclusively, the routine performance of SB pretreatment before the PS procedures was not recommended by the current consortium (24) and previous clinical trials (4–9). SB pretreatment should be avoided for simple bifurcation lesions (short lesion length and less severe disease in the SB).

Acute coronary syndrome is a stage where coronary plaques become unstable (25). The COBIS Registry showed a lower rate of 3-year TLF after the PS approach for patients with ACS but no difference between the PS and upfront 2-stent for patients without ACS (26), confirmed by another study of Korean team which further found that SB lesion length was an independent factor of TLF (27). The underlying mechanisms for a higher rate of TLF in patients with ACS were multifactorial, of them DAPT might be one major reason (25). However, this study reported more frequent prescription of DAPT in the PS with 2-stent group and DAPT was not the factor for predicting TLF. This may again address the critical importance of intravascular imaging in guiding SB treatment.

### Limitations

This study has some limitations. First, the non-randomized feature raised concerns about the exact different treatment effects between the PS with 1-stent and the PS with 2-stent as SB rescue stenting was performed in the scenario that SB was severely compromised or had complications induced by pretreatment. Thus, a randomized study using physiological assessment as the sole criterion for treating SB after stenting MV is crucial. Second, we did not compare the difference in clinical outcome between the PS with T and the PS with T-and-Protrusion (TAP) (4, 9, 10, 24) when a second stent was required in the SB. When an SB needed to be dilated after MV stenting, rewire was recommended across the distal cell of the MV stent (24). However, a very narrow space at the ostial SB after stenting MV did not allow precisely rewiring (from proximal or distal cell) so long as successfully crosses the struts to restore the SB flow, particularly for complex bifurcations (4). Finally, intravascular imaging was not routinely used because imaging guidance was not recommended in the studies (4, 9–11). Therefore, an intravascular imaging-guided stenting bifurcation is highly recommended. For this issue, two ongoing studies (28, 29) would launch their results, demonstrating the advantage of intravascular imaging guidance in treating a bifurcation lesion.

## Conclusion

Provisional stenting with 2-stent is associated with a higher rate of 3-year TLF, mainly due to increased requirement of revascularization. A further study identifying the underlying mechanisms correlated with stent failure is warranted.

## Data availability statement

The raw data supporting the conclusions of this article will be made available by the authors, without undue reservation.

## Ethics statement

The studies involving human participants were reviewed and approved by Ethics Committee of Nanjing First Hospital. The patients/participants provided their written informed consent to participate in this study.

## Author contributions

S-LC and IS made substantial contributions to the initial conception and design of the whole study and to the revision of the manuscript. IS and ZG wrote the first draft. JK contributed to data management and statistical expertise. J-JZ, TS, MM, FY, and NT provided comments and suggestions in critical revision of the manuscript. All authors approved the final version of the article.

## Abbreviations

PS: provisional stenting
ACS: acute coronary syndrome
TLF: target lesion failure
TVF: target vessel failure
TLR: target lesion revascularization
SB: side branch
MV: main vessel
DK crush: double kissing crush
KBI: kissing balloon inflation
TIMI: thrombolysis in myocardial infarction
POT: proximal optimization technique
QCA: quantitative coronary analysis
TVMI: target vessel myocardial infarction
ST: stent thrombus
DAPT: dual antiplatelet therapy
PMI: periprocedural myocardial infarction
PCI: percutaneous coronary intervention
CABG: coronary artery bypass graft.
