# Supplementary material for: Provisional stenting with side branch rescue stenting is associated with increased 3-year target lesion failure in patients with acute coronary syndrome and coronary bifurcation lesions
Source: Front Cardiovasc Med. 2022 Oct 11;9:910313. doi: 10.3389/fcvm.2022.910313 (PMC9595024; doi:10.3389/fcvm.2022.910313)
Supplement: Supplementary file 1 [file Data_Sheet_1.docx]

**Supplemental materials**

**Table S 1. Comparison between with and without pretreatment**

|  | SB Pretreatment  (n=338) | SB no pretreatment (n=482) | p |
| --- | --- | --- | --- |
| **QCA variables** | | | |
| SB lesion length, mm | 14.47±9.91 | 12.25±8.65 | 0.001 |
| Ostial SB RVD, mm | 2.36±0.45 | 2.43±0.43 | 0.036 |
| SB MLD, mm | 1.08±0.53 | 1.36±0.54 | <0.001 |
| Ostial SB DS, % | 51.99±20.16 | 42.96±19.26 | <0.001 |
| **Clinical outcome** | | | |
| **30 days, n (%)**  TLF  Cardiac death  TVMI  PMI  TLR  Stent thrombosis | 31 (9.2)  4 (1.2)  29 (8.6)  15 (4.4)  2 (0.6)  7 (2.1) | 27 (5.6)  2 (0.4)  23 (4.8)  9 (1.9)  3 (0.6)  4 (0.8) | 0.054  0.236  0.030  0.036  1.000  0.215 |
| **1-year, n (%)**  TLF  Cardiac death  TVMI  TLR  Stent thrombosis | 64 (18.9)  9 (2.7)  33 (9.8)  30 (8.9)  9 (2.7) | 54 (11.2)  7 (1.5)  29 (6.0)  28 (5.8)  12 (2.5) | 0.002  0.305  0.060  0.098  1.000 |
| **3-year, n (%)**  TLF  Cardiac death  TVMI  TLR  Stent thrombosis | 83 (24.6)  18 (5.3)  41 (12.1)  47 (13.9)  16 (4.7) | 71 (14.7)  13 (2.7)  36 (7.5)  35 (7.3)  16 (3.3) | 0.001  0.063  0.028  0.002  0.360 |

DS, diameter stenosis; MLD, minimal lumen diameter; PMI, periprocedural myocardial infarction; QCA, quantitative coronary analysis; SB, side branch; RVD, reference vessel diameter; TLF, target lesion failure; TLR, target lesion revascularization; TVMI, target vessel myocardial infarction


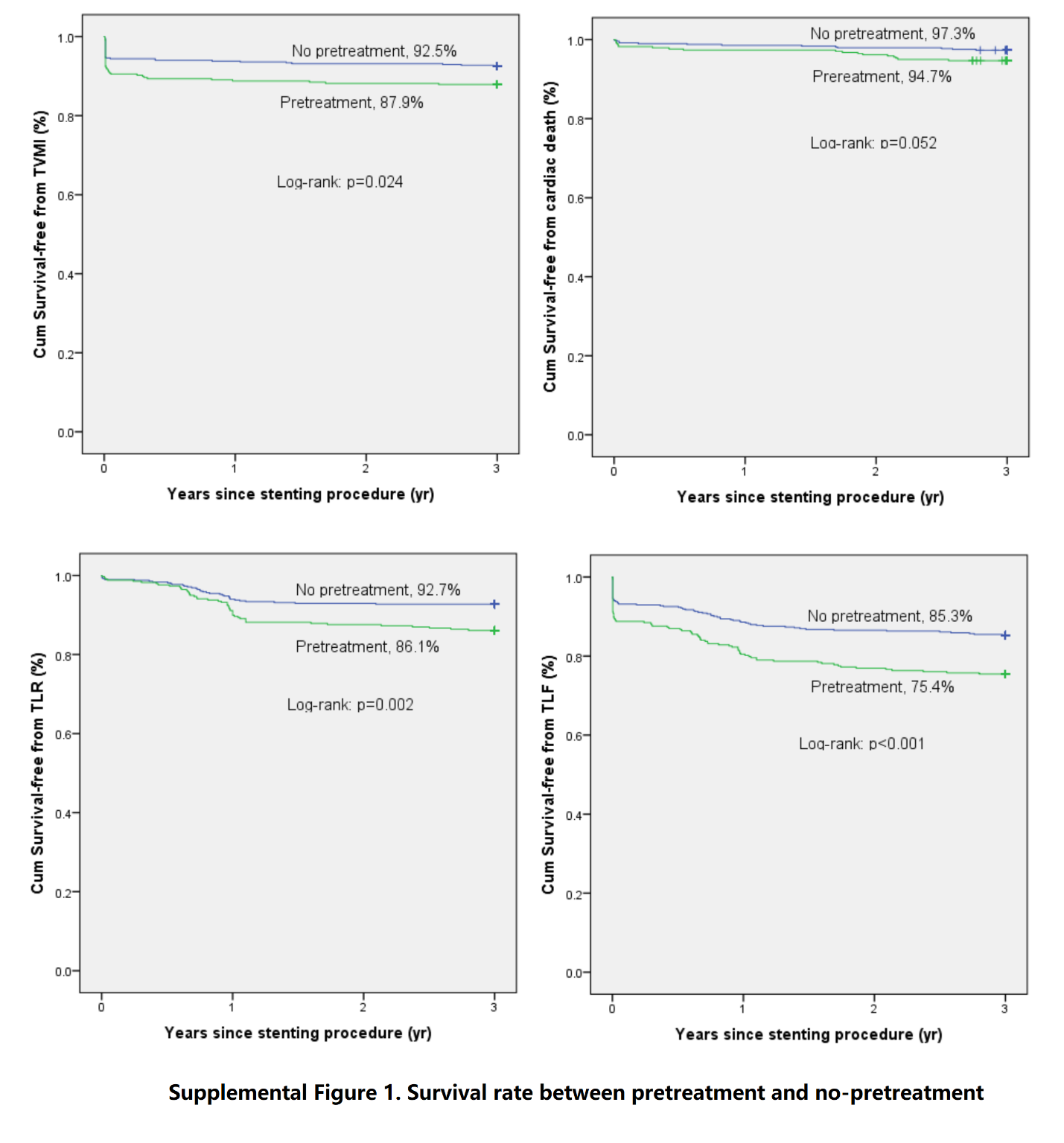
 TLF, target lesion failure; TLR, target lesion revascularization; TVMI, target vessel myocardial infarction
